# Change in body weight and risk of hypertension after switching from efavirenz to dolutegravir in adults living with HIV: evidence from routine care in Johannesburg, South Africa

**DOI:** 10.1016/j.eclinm.2023.101836

**Published:** 2023-02-06

**Authors:** Alana T. Brennan, Cornelius Nattey, Emma M. Kileel, Sydney Rosen, Mhairi Maskew, Andrew C. Stokes, Matthew P. Fox, Willem D.F. Venter

**Affiliations:** a Health Economics and Epidemiology Research Office, Faculty of Health Sciences, University of the Witwatersrand, Johannesburg, South Africa; b Department of Global Health, Boston University School of Public Health, Boston, MA, USA; c Department of Epidemiology, Boston University School of Public Health, Boston, MA, USA; d Ezintsha, Faculty of Health Sciences, University of the Witwatersrand, Johannesburg, South Africa

**Keywords:** Integrase strand transfer inhibitors, Dolutegravir, Efavirenz, Weight, Hypertension, South Africa

## Abstract

**Background:**

The integrase strand transfer inhibitor (INSTI) dolutegravir is recommended in World Health Organization guidelines, but is associated with weight gain. We evaluated weight change in patients switching from efavirenz to dolutegravir in first-line antiretroviral therapy (ART) in Johannesburg, South Africa.

**Methods:**

We conducted a prospective cohort study of adults (≥16 years) of black African ancestry with HIV who initiated ART between January 2010–December 2020. Patients were propensity score-matched 1:1 (unexposed i.e. remaining on efavirenz: exposed i.e. switched from efavirenz to dolutegravir) on sex, age, months on ART, first ART regimen, haemoglobin, body mass index (BMI), blood pressure, viral load and CD4 count. We used linear regression to assess the effect of switching from efavirenz to dolutegravir on weight change and hypertension 12 months after exposure.

**Findings:**

We matched 794 patients switching to dolutegravir to 794 remaining on efavirenz. Exposed patients had a higher mean change in weight (1.78 kg; 95% confidence interval (CI):1.04,2.52 kg) from start of follow-up to 12 months vs. unexposed. We also found a 14.2 percentage point increase (95% CI: 10.6,17.7) in the risk of hypertension in those exposed to dolutegravir vs those that remained on efavirenz.

**Interpretation:**

In a real-world population, patients gained more weight and were at higher risk of hypertension after switching from efavirenz to dolutegravir than those remaining on efavirenz. Longer follow-up is needed, however, to determine if INSTI-associated weight gain is associated with changes in non-communicable disease risk over the long-term, or whether weight gain is sustained, as seen in clinical trials.

**Funding:**

This study has been made possible by the generous support of the American People and the President's Emergency Plan for AIDS Relief (PEPFAR) through the 10.13039/100000200United States Agency for International Development (USAID), under the terms of cooperative agreement cooperative Agreement 72067419CA00004. In addition to the 10.13039/100000062National Institute of Diabetes and Digestive and Kidney Diseases (NIDDK) 1K01MH105320-01A1.

**Research in context**

**Evidence before this study**

In 2016, World Health Organization guidelines recommended integrase strand transfer inhibitors (INSTIs), specifically dolutegravir, as an alternative to efavirenz in first-line antiretroviral therapy (ART), with an updated recommendation including it as the preferred drug over efavirenz from 2018. The recommendation reflected the INSTI dolutegravir's favourable profile with regard to sustained viral suppression, resistance, and tolerance over efavirenz. However, in 2020, results of two large randomized clinical trials out of sub-Saharan Africa demonstrated substantial weight gain among people taking dolutegravir compared to efavirenz, The New Antiretroviral and Monitoring Strategies in HIV-infected Adults in Low-income countries (NAMSAL) trial and The ADVANCE trial. In sub-Saharan Africa, where the prevalence of obesity and uptake of ART are simultaneously on the rise, examining ART-associated weight gain and subsequent cardiometabolic risks in routine care, is of the utmost importance.

**Added value of this study**

We sought to compare absolute and percentage weight change and risk of hypertension over 12 months between people living with HIV (PLWH) in Johannesburg, South Africa who switched from efavirenz to dolutegravir in their standard first-line regimen to PLWH who remained on efavirenz. We conducted a prospective cohort study where we matched 794 patients switching to dolutegravir to 794 patients remaining on efavirenz. Our data confirm findings from these previous clinical trials and demonstrate that in a real-world population of PLWH on ART, patients gained 1.78 kg in weight 12 months post switching from efavirenz to dolutegravir than those remaining on efavirenz. We also saw a 14.2 percentage point increase in the risk of hypertension in those exposed to dolutegravir compared to those that remained on efavirenz.

**Implications of all the available evidence**

Dolutegravir will continue to be the drug of choice in first-line ART in low- and middle-income countries (LMICs) due to its cost-savings potential, better tolerability—leading to better retention in care and higher potency—leading to fewer people needing to switch to costlier second-line regimens. However, long-term follow-up is needed in clinical trials and from routine care to determine if dolutegravir-associated weight gain is sustained over time and if the weight gain is associated with changes in non-communicable diseases (NCD) (e.g., hypertension, type 2 diabetes mellitus) risk.

## Introduction

In 2016, World Health Organization (WHO) guidelines recommended integrase strand transfer inhibitors (INSTIs), specifically dolutegravir, as an alternative to efavirenz in first-line antiretroviral therapy (ART), with an updated recommendation including it as the preferred drug over efavirenz from 2018.^1^ The recommendation reflected the INSTI dolutegravir's favourable profile with regard to sustained viral suppression, resistance, and tolerance over efavirenz. The Clinton Health Access Initiative estimates that 90% of first-line antiretroviral patients in low- and middle-income countries (LMICs) will be taking dolutegravir-containing fixed dose combinations and suggests that switching from efavirenz to dolutegravir will improve patient outcomes and ultimately lead to cost savings.^2^

As the virus itself causes weight loss in people living with HIV (PLWH), many patients gain weight following ART initiation as part of their ‘return to health’. Such weight gain has been shown to have positive effects on survival, particularly in those underweight at the start of ART, particularly in LMICs where wasting is more prevalent and treatment initiation is often delayed to more advanced stages of HIV disease.^3^^,^^4^ The benefits of weight gain, however, may be diminishing due to steadily rising rates of obesity globally and the association of obesity with an increased risk of mortality. For patients who are overweight at the time of ART initiation and who start ART at an earlier stage of HIV disease, a return to their pre-HIV weight may in fact create additional health risks. ART-associated weight gain among PLWH could result in incident obesity and subsequent increased risk of metabolic and cardiovascular diseases.^5^^,^^6^

Potentially worsening this risk, two large randomised clinical trials in sub-Saharan Africa have demonstrated substantial weight gain among people taking dolutegravir compared to efavirenz. The New Antiretroviral and Monitoring Strategies in HIV-infected Adults in Low-income countries (NAMSAL) study, conducted in Cameroon, randomized 613 ART naïve participants to dolutegravir or efavirenz at the low dose of 400 mg combined with tenofovir disoproxil fumarate (tenofovir) and lamivudine. The study reported greater increases in body weight and treatment-emergent obesity in the dolutegravir arm.^7^ The ADVANCE trial, conducted in South Africa, randomized 1053 ART-naive participants to dolutegravir or efavirenz at the standard dose of 600 mg, both combined with emtricitabine and tenofovir, and a third arm of dolutegravir combined with emtricitabine and tenofovir alafenamide (TAF). Results showed greater increases in body weight in both dolutegravir arms compared to efavirenz.^8^

Earlier studies evaluating the impact of INSTIs on long-term weight have found that most weight gain occurs within the first 24 months following initiation, then plateaus,^5^ while recent results from the ADVANCE trial show weight gain continues to be higher in patients on dolutegravir through 196 weeks of follow-up.^9^ The magnitude of short-term weight gain (48 weeks) in the dolutegravir arms of the ADVANCE and NAMSAL trials, particularly among female participants, remains cause for concern, as substantial weight gain and incident obesity are major risk factors for multiple non-communicable diseases (NCDs), such as diabetes and hypertension.^10^ In sub-Saharan Africa, where the prevalence of obesity and uptake of ART are simultaneously on the rise, examining ART-associated weight gain and subsequent cardiometabolic risks in routine care, is of the utmost importance. We therefore compared absolute and percentage weight change and risk of hypertension over 12 months between PLWH in Johannesburg, South Africa who switched from efavirenz to dolutegravir in their standard first-line regimen to PLWH who remained on efavirenz.

## Methods

### Cohort description

**Fig. 1:**
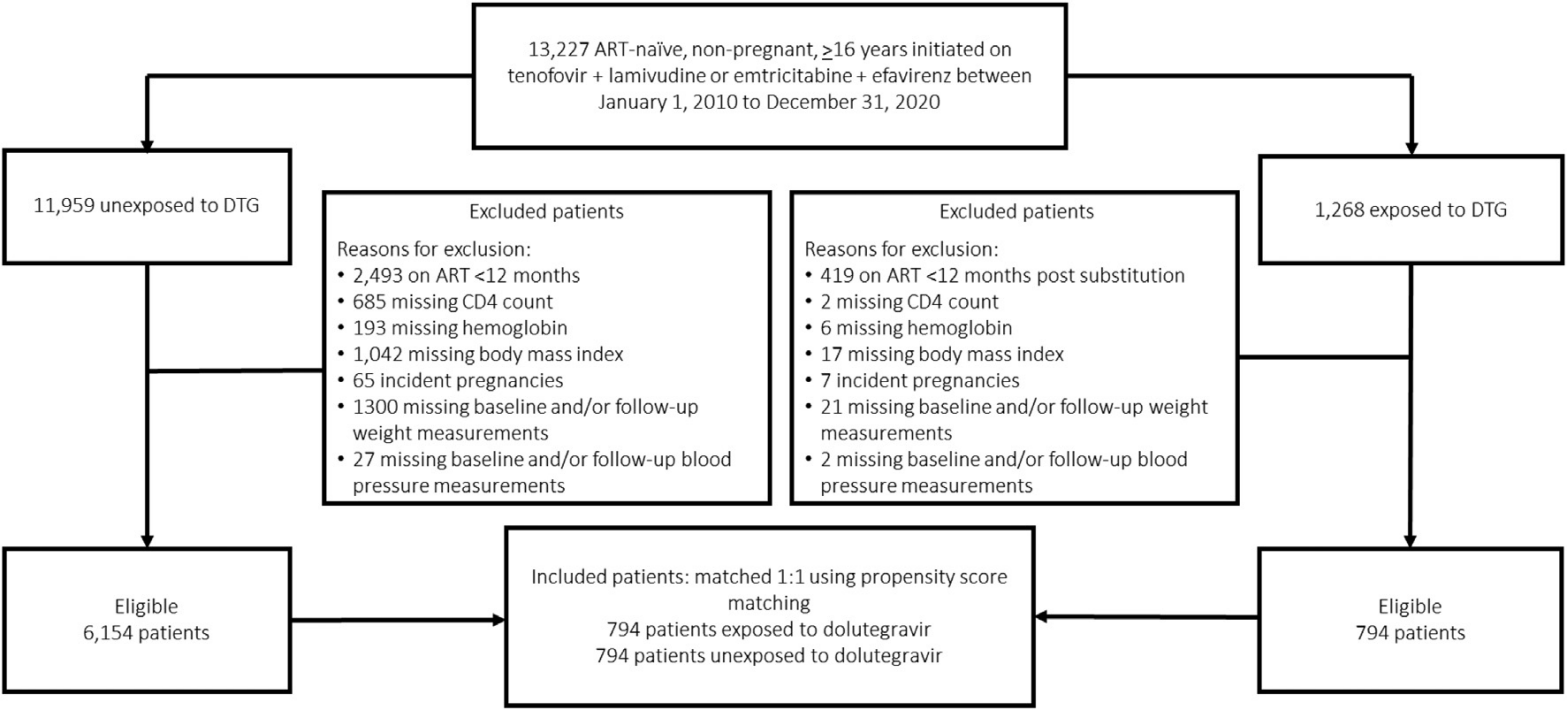
**Selection of study patients for a study of the effects of dolutegravir vs. efavirenz in people living with HIV in Johannesburg, South Africa (n = 13,227)**.

We conducted a matched prospective cohort study amongst ART-naïve, non-pregnant adults (≥16 years) living with HIV who initiated a regimen of tenofovir disoproxil fumarate (tenofovir), efavirenz, and lamivudine or emtricitabine, which were the standard first-line regimens during our study period, between January 1, 2010 and December 31, 2020 for patients in the Themba Lethu clinical cohort in Johannesburg, South Africa.^11^ Tenofovir alafenamide was not used at the clinic during this time. We excluded patients who were missing a weight at the start of follow-up and/or 12-month follow-up weight (Fig. 1). We also excluded patients with any of the following missing data needed to calculate the propensity score: CD4 count, haemoglobin, body mass index (BMI), systolic or diastolic blood pressure around the time of switch (interchangeable with ‘baseline’ throughout the manuscript) (Fig. 1).

Themba Lethu Clinic, which is located at a large urban secondary hospital, began initiating patients onto ART in 2004 under South Africa's public-sector treatment program. Care and treatment are provided according to national treatment guidelines.^12^ Dolutegravir became available to patients at the clinic in 2019. As of December 2021, 4374 patients were actively receiving treatment for HIV at the clinic. Patient data, including demographic characteristics, clinical conditions, laboratory test results, and medications, are entered into an electronic patient management system, TherapyEdge-HIV™, by a clinician or a data entry clerk. Weight, height, and blood pressure are routinely measured at medical visits. Patients are typically seen for medical follow-up visits at months 1, 3, and 6 and 12-monthly thereafter. Patients come for HIV drug pickups monthly for the first 6–12 months on treatment and every 2 months thereafter once stable.

Starting in 2019, patients were switched from the fixed dose combination containing efavirenz to that containing dolutegravir at their regular visits. National guidelines at the time suggested a switch if a viral load was undetectable and taken in the last 6 months.^12^ However, clinicians at the site indicate that at the time the patients in our analysis were being switch, there was no priority setting with the Department of Health in the transition plan, for any particular group, nor was there any clear group of patients that clinicians would naturally prioritize. We speculate that the transition was driven more by motivated clinicians within the clinic being on duty at any one time, as probably the most important factor.

Exposed individuals in our study were defined as patients who switch from efavirenz to dolutegravir, while unexposed patients were those that remained on their initial standard first line regimen containing efavirenz over the course of follow-up. Tenofovir plus lamivudine or emtricitabine remained the other two drugs in all patient's regimens for the entire study period, the only change that was allowed in the analysis was efavirenz for dolutegravir.

### Propensity score matching

Since we assume that switching from efavirenz to dolutegravir was not randomly assigned, we used propensity score matching to create similar populations of exposed and unexposed patients^13^ (Supplemental Fig. S1). We used the psmatch procedure with a caliper of 0.00 (STATA v. 15). The psmatch procedure uses a logistic regression model to compute propensity scores used for matching. We predicted exposure as a function of biological sex and age at ART initiation (categorized as 16–24.9, 25–29.9, 30–39.9, 40–49.9, and ≥50 years), in addition to the following variables at time of switch time on ART (categorized as 0–11.9, 12–23.9, 24–35.9, 36–47.9, 48–59.9, and ≥60 months), first ART regimen (emtricitabine vs. lamivudine as all patients were initiated onto a regimen containing tenofovir), haemoglobin (<10 vs. ≥10 ug/dL), CD4 count (categorized as <200, 200–299, 300–399, 400–499 and ≥ 500 cells/mm^3^), BMI (<18.5, 18.5–24.9, 25–29.9, ≥30 kg/m^2^), viral load suppression (<1000 copies/mL) and hypertension (defined as having a diastolic blood pressure >90 mmHg AND/OR systolic blood pressure >140 mmHg^14^ or being prescribed anti-hypertension medication (i.e., amlodipine besylate, benazepril hydrochloride, candesartan cilexetil, captopril, enalapril maleate, enalaprilat, felodipine, lisinopril, losartan potassium, nifedipine, nisoldipine, olmesartan medoxomil, perindopril erbumine, perindopril terbutylamine, quinapril hydrochloride, ramipril, telmisartan, valsartan or verapamil hydrochloride)). The anti-hypertensive medication start date had to be prior to a patients baseline date with no end date documented because the patient was still actively taking the medication, or the start date had to be prior to the baseline date with an end date that fell on or after the baseline date, indicating the patient was on the medication at baseline. We matched 1 exposed to 1 unexposed patient without replacement.

While anti-hypertensive medications may plausibly be used for other indications (e.g., angiotensin-converting enzyme (ACE)-inhibitors for proteinuria alone, verapamil for arrythmias), clinicians at the site indicate this is uncommon. As such, for the purposes of this analysis, we have assumed commonly prescribed anti-hypertensive drug classes, largely ACE-inhibitors, angiotensin II receptor antagonists, thiazide diuretics and calcium-channel blockers, as indications of diagnosed hypertension. Additionally, we note that a single measurement of systolic and diastolic blood pressure, such as what we used as part of the hypertension definition for this study, does not confirm a diagnosis of hypertension according to local primary health care (PHC) guidelines.^14^

### Follow up time

For those who switched to dolutegravir, follow-up time began at the date of the switch. Those remaining on efavirenz do not have an event time to distinguish the start of follow up. As such, for those who remained on efavirenz, follow up time began at the date that the person they were matched to switched to dolutegravir. For example, if a person switched to dolutegravir after 15 months on efavirenz then both this exposed person and their unexposed match would start follow up time at 15 months on ART.

### Outcomes

Weight outcomes were defined as:

*Baseline weight* was defined as the closest weight recorded 6 months prior to the date of switch for those in the dolutegravir group or the equivalent matched baseline date for those remaining on efavirenz.

*Follow-up weight* was defined 12 months after the start of follow up with the closest weight recorded 6 months prior to that 12-month end point.

*Change in weight* was calculated by simply subtracting the 12-month follow-up weight from baseline weight.

*Percent change in weight* was calculated by taking the change in weight between the two time points divided by the baseline weight.

*Hypertension at* 12-months was defined as either being prescribed anti-hypertension medication one-month post baseline date up to 12-months or having a diastolic blood pressure >90 mmHg AND/OR a systolic blood pressure >140 mmHg^14^ 6 months prior to the 12-month end point.

### Statistical analysis

We used simple proportions to describe categorical data for the characteristics of the cohort prior to matching that were included in the propensity score. For the final matched cohort, medians with interquartile ranges (IQR) and simple proportions were used to describe continuous and categorical data, respectively. We used crude and adjusted linear ordinary least squares regression to model outcomes (mean change in weight, percent change in weight and hypertension) as a function of sex, age at ART initiation, time on ART, BMI, emtricitabine vs. lamivudine, haemoglobin, CD4 count, hypertension, viral load status and number of days between date of baseline weight and time of switch and days between follow-up weight and 12-months. We added interaction terms between all variables in our model and exposure groups to determine if any variables modified the relationship between dolutegravir and the outcome of mean change in weight. As none of these were prespecified a priori and given our limited sample size we consider these as hypothesis generating only. While the distribution of our data on weight was slightly right-skewed, we believe it was still appropriate to report means, as the median and mean were almost overlapping.

### Ethics statement

Use of data was approved by the Human Research Ethics Committee of the University of the Witwatersrand (M140201). Approval for analysis of de-identified data was granted by the Institutional Review Board of Boston University (H-29768). Data on patients from Themba Lethu Clinic was collected for routine clinical purposes and not for research purposes. We had no direct contact with patients, as such, informed consent was not obtained from subjects. An inform consent waiver was received from the Boston University's Institutional Review Board and the Human Research Ethics Committee of the University of the Witwatersrand.

### Role of funding source

The contents are the responsibility of the authors and do not necessarily reflect the views of the NIDDK. The funders had no role in the study design, data collection and analysis, decision to publish, or preparation of the manuscript. The corresponding author had final responsibility for the decision to submit for publication.

## Results

A total of 13,227 ART-naïve, non-pregnant, ≥16 years initiated on tenofovir + lamivudine or emtricitabine + efavirenz between January 1, 2010 to December 31, 2020 patients were eligible for our analysis, including 11,959 unexposed and 1268 exposed patients (Fig. 1). After removing those with missing clinical data, not on ART for at least 12-months and incident pregnancies, 6154 unexposed and 794 exposed patients were eligible for our matched analysis (Table 1). Males (vs. female), those <25 years of age (vs. ≥25 years), those on ART longer, patients with higher haemoglobin (≥10.0 vs. <10.0 ug/dL), those with a BMI ≥18.5 kg/m^2^ (vs. <18.5 kg/m^2^), patients virally suppressed (vs. unsuppressed) and those on a regimen containing lamivudine (vs. emtricitabine) had an increased probability of switching from efavirenz to dolutegravir, as shown in Table 1.

**Table 1:** Characteristics of study cohort and predictors of switching from efavirenz to dolutegravir (exposed) vs remaining on efavirenz (unexposed) among ART patients at the Themba Lethu Clinic in Johannesburg South Africa (n = 6948).

Variable at time of switch	Unexposed (n = 6154)	Exposed (n = 794)	Adjusted Odds Ratio^a^ (95% CI)
**Sex of patients**			
Female	3819 (90.9)	384 (9.14)	**ref.**
Male	2335 (85.1)	410 (14.9)	1.94 (1.64, 2.31)
**Age (years) at ART initiation**			
18–24.9	343 (76.9)	103 (23.1)	2.50 (1.80, 3.48)
25–29.9	781 (93.4)	55 (6.58)	0.54 (0.38, 0.77)
30–39.9	2531 (90.5)	265 (9.48)	0.71 (0.56, 0.92)
40–49.9	1776 (87.4)	256 (12.6)	0.89 (0.69, 1.15)
≥50	723 (86.3)	115 (13.7)	**ref.**
**CD4 count (cells/mm**^**3**^**) at time of switch**			
<200	585 (90.3)	63 (9.72)	**ref.**
200–299	823 (87.2)	121 (12.8)	1.05 (0.74, 1.49)
300–399	1067 (88.7)	136 (11.3)	0.86 (0.61, 1.22)
400–499	1022 (89.3)	122 (10.7)	0.76 (0.53, 1.08)
≥500	2657 (88.3)	352 (11.7)	0.82 (0.60, 1.14)
**Viral load suppression (<1000 copies/mL)**			
No	319 (98.2)	6 (1.85)	**ref.**
Yes	5819 (88.1)	788 (11.9)	6.86 (0.82, 1.27)
**Time (months) on ART at time of switch**			
0–11.9	1159 (92.9)	89 (7.13)	**ref.**
12–23.9	917 (86.2)	147 (13.8)	2.03 (1.52, 2.70)
24–35.9	771 (88.6)	99 (11.4)	1.55 (1.14, 2.13)
36–47.9	643 (89.3)	77 (10.7)	1.57 (1.12, 2.20)
48–59.0	686 (90.6)	71 (9.38)	2.45 (1.72, 3.48)
≥60	1978 (86.4)	311 (13.6)	8.85 (6.48, 12.1)
**Haemoglobin (ug/dL) at time of switch**			
<10.0	261 (94.6)	15 (5.43)	**ref.**
≥10.0	5896 (88.3)	779 (11.7)	1.44 (0.83, 2.51)
**Body mass Index (kg/m**^**2**^**) at time of switch**			
<18.5	413 (94.3)	25 (5.71)	**ref.**
18.5–24.9	2449 (89.6)	285 (10.4)	1.67 (1.08, 2.59)
25–29.9	1701 (88.3)	225 (11.7)	2.13 (1.36, 3.34)
30–39.9	1591 (86.0)	259 (14.0)	3.03 (1.93, 4.76)
**Hypertension at time of switch**			
No	5271 (88.8)	663 (11.2)	**ref.**
Yes	883 (87.1)	131 (12.9)	1.02 (0.82, 1.27)
**Nucleoside reverse transcriptase inhibitor**			
emtricitabine	3322 (94.0)	213 (6.03)	**ref.**
lamivudine	2832 (83.0)	581 (17.0)	8.61 (6.80, 10.9)

a Odds ratios calculated from a logistic regression model; STATA (v. 15.0) psmatch procedure was used to create propensity scores to match patients for analyses of our outcomes.

When comparing demographic and clinical characteristics at ART initiation of those excluded to those included in the final dataset used to create the propensity score used for matching, we found that they were comparable on all observed covariates, median age (excluded 36.7 years; interquartile range (IQR): 30.3, 44.1 years vs. included 37.9 years; IQR: 31.9, 44.7 years), median haemoglobin (excluded 12.0 ug/dL; IQR: 10.3, 13.5 ug/dL vs. included 12.4 ug/dL; IQR: 10.9, 13.8 ug/dL), proportion female (excluded 56.0% vs. included 60.5%), median CD4 count (excluded 143 cells/mm^3^; IQR: 47, 273 cells/mm^3^ vs. included 170 cells/mm^3^, IQR 69, 279 cells/mm^3^) and WHO stage 3/4 conditions (excluded 23.0% vs. included 17.6%) at ART initiation.

### Matched cohort

Our propensity score matching of the 794 dolutegravir exposed patients to 794 patients unexposed to dolutegravir created similar populations with regards to demographic and clinical characteristics (Table 2). The 1588 patients included in the matched analysis were predominately initiated onto a standard first line regimen containing lamivudine (72.6%). Just over half (51.5%) were male with a median age of 38.8 years (IQR: 31.7, 45.8 years). Overall the population had a median CD4 cell count of 470 cell/mm^3^ (IQR: 319, 631 cell/mm^3^), a median haemoglobin of 14.0 ug/dL (IQR: 12.8, 15.2 ug/dL), a median time on ART of 43.4 months (IQR: 20.1, 74.8 months), a median systolic blood pressure of 130 mmHG (IQR: 120.0, 140.0 mmHG), and a median diastolic blood pressure of 81 mm/HG (IQR: 74.0, 89.0). Less than 1% of patients had a detectable viral load at baseline and at 12-months.

**Table 2:** Patient demographic and characteristics at time of switch among stable patients on HIV treatment at the Themba Lethu Clinic in Johannesburg South Africa after propensity score matching (n = 1588).

	DTG unexposed (n = 794)	DTG exposed (n = 794)	Total (n = 1588)
**Sex** (%)			
Female	387 (48.7)	384 (48.4)	771 (48.6)
Male	407 (51.3)	410 (51.6)	817 (51.5)
**Age (years)** at ART initiation (%)			
16–24.9	113 (14.2)	103 (13.0)	216 (13.6)
25–29.9	52 (6.55)	55 (6.93)	107 (6.74)
30–39.9	277 (34.9)	265 (33.4)	542 (34.1)
40–49.9	237 (29.8)	256 (32.2)	493 (31.1)
≥50	115 (14.5)	115 (14.5)	230 (14.5)
**Age** (median (IQR))	28.6 (31.8, 45.7)	39.0 (31.7, 45.8)	38.8 (31.7, 45.8)
**CD4 count (cells/mm**^**3**^**) at time of switch** (%)			
<200	51 (6.42)	63 (7.93)	114 (7.18)
200–299	115 (14.5)	121 (15.2)	236 (14.9)
300–399	136 (17.1)	136 (17.1)	272 (17.1)
400–499	128 (16.1)	122 (15.4)	250 (15.7)
≥500	364 (45.8)	352 (44.3)	716 (45.1)
**CD4 count** (median (IQR))	473 (329, 621)	470 (314, 642)	470 (319, 631)
**Time on ART (months) at time of switch** (%)			
0–11.9	90 (11.3)	89 (11.2)	179 (11.3)
12–23.9	165 (20.8)	147 (18.5)	312 (19.7)
24–35.9	100 (12.6)	99 (12.5)	199 (12.5)
36–47.9	71 (8.94)	77 (9.70)	148 (9.32)
48–59.9	67 (8.44)	71 (8.94)	138 (8.69)
≥60	301 (37.9)	311 (39.2)	612 (38.5)
**Time on ART** (median (IQR))	42.2 (19.9, 70.6)	45.4 (20.2, 86.5)	43.4 (20.1, 74.8)
**Body Mass Index at time of switch** (kg/m^2^) (%)			
<18.5	21 (2.64)	25 (3.15)	46 (2.90)
18.5–24.9	281 (35.4)	285 (35.9)	566 (35.6)
25–29.9	214 (27.0)	225 (28.3)	439 (27.6)
≥30.0	278 (35.0)	259 (32.6)	567 (33.8)
**Body Mass Index** (median (IQR))	26.6 (23.0, 31.3)	27.0 (22.8, 31.5)	27.0 (22.9, 31.4)
**Haemoglobin (ug/dL) at time of switch** (%)			
<10.0	10 (1.26)	15 (1.89)	25 (1.57)
≥10.0	784 (98.7)	779 (98.1)	1563 (98.4)
**Haemoglobin** (median (IQR))	14.0 (12.9, 15.4)	14.0 (12.7, 15.1)	14.0 (12.8, 15.2)
**Nucleoside reverse transcriptase inhibitor** (%)			
lamivudine	572 (72.0)	581 (73.2)	1153 (72.6)
emtricitabine	333 (28.0)	213 (26.8)	435 (27.4)
**Unsuppressed viral load** (%)	8 (1.01)	6 (0.76)	14 (0.90)
**Systolic (mm/Hg)** (median (IQR))	128 (119, 139)	133 (122, 141)	130 (120, 140)
**Diastolic (mm/Hg)** (median (IQR))	80 (72, 87)	83 (75, 90)	81 (74, 89)
**Hypertension at time of switch** (%)	128 (16.1)	132 (16.6)	260 (16.4)
**12-month outcomes**			
**Unsuppressed viral load** (%)	8 (1.01)	4 (0.50)	12 (0.76)
**Hypertension** (%)	87 (11.0)	201 (25.3)	288 (18.1)
**Baseline weight (kg)** (mean (SD))	74.0 (15.8)	73.1 (16.9)	73.5 (16.4)
**Follow-up weight (kg)** (mean (SD))	75.4 (16.3)	75.9 (17.0)	75.7 (16.6)
**Mean change in weight (kg)**	1.5 (1.3)	2.8 (6.7)	2.12 (6.0)
**Percent change in weight** (%)	2.2 (7.6)	4.5 (12.2)	3.3 (10.2)

### Association of switching from efavirenz to dolutegravir with weight change over 12 months

Baseline weights in kilograms (kg) were comparable between our efavirenz (mean 74.0 kg; standard deviation (SD): 15.8 kg) and dolutegravir exposed groups (mean 73.1 kg; SD: 16.9 kg) (Table 2). For efavirenz patients, mean weight from at 12 months increased to 75.4 kg (SD: 16.3 kg), corresponding to a mean change in weight of 1.5 kg (SD: 5.2 kg). For dolutegravir exposed patients, mean weight at 12 months increased to 75.9 kg (SD: 17.0 kg), corresponding to a mean change in weight of 2.8 kg (SD: 6.7 kg). This represents a crude percentage weight increase of 2.2% (SD: 7.6) for efavirenz patients and 4.5% (SD: 12.2) for those exposed to dolutegravir.

Our results remained consistent in linear regression models adjusted for sex, age, time on ART, BMI, emtricitabine vs. lamivudine, haemoglobin, viral load status and CD4 count. Patients who switched to dolutegravir had a higher mean weight change of 1.78 kg (95% confidence interval (CI): 1.04, 2.52 kg) and a higher percent change in weight of 2.90% (95% CI: 1.63, 4.17%) from baseline to 12 months when compared to those remaining on efavirenz (Table 3). Our results also suggest that independent of dolutegravir exposure status, patients with lower BMI (<30 vs.≥30), those <50 years (vs. ≥50 years) and patients on treatment <12 months (vs.≥60 months) experienced a higher mean and percent change in weight over the 12-month follow-up period.

**Table 3:** Crude and adjusted linear regression models of predictors of mean change and percent change in weight and hypertension at 12-months among patients on HIV treatment at the Themba Lethu Clinic in Johannesburg South Africa (n = 1588).

	Crude mean change in weight∗ (95% CI)	Adjusted mean change in weight∗ (95% CI)	Crude percent change in weight∗ (95% CI)	Adjusted percent change in weight∗ (95% CI)	Crude hypertension (RD; 95% CI)	Adjusted hypertension (RD; 95% CI)
**Exposed**						
efavirenz	**ref.**	**ref.**	**ref.**	**ref.**	**ref.**	**ref.**
dolutegravir	1.34 (0.75, 1.93)	1.78 (1.04, 2.52)	2.31 (1.31, 3.31)	2.90 (1.63, 4.17)	14.4 (10.6, 18.1)	14.2 (10.6, 17.7)
**Age at ART initiation (years)**						
16–24.9	−0.10 (−1.22, 1.02)	0.77 (−0.37, 1.91)	0.36 (−1.54, 2.26)	1.43 (−0.52, 3.39)	−23.1 (−30.1, −16.1)	−14.2 (−21.2, −7.19)
25–29.9	0.92 (−0.46, 2.30)	1.08 (−0.29, 2.44)	1.24 (−1.10, 3.59)	1.25 (−1.11, 3.60)	−22.1 (−30.8, −13.4)	−15.0 (−23.4, −6.55)
30–39.9	0.54 (−0.39–1.47)	0.61 (−0.30, 1.52)	0.57 (−1.01, 2.15)	0.65 (−0.91, 2.22)	−14.4 (−20.3, −8.60)	−9.61 (−15.2, −4.00)
40–49.9	0.69 (−0.26, 1.63)	0.69 (−0.23, 1.61)	1.06 (−0.54, 2.66)	1.06 (−0.52, 2.64)	−6.03 (−12.0, −0.11)	−3.30 (−8.96, 2.37)
≥50	**ref.**	**ref.**	**ref.**	**ref.**	**ref.**	**ref.**
**CD4 Category (cells/mm**^**3**^**)**						
<200	**ref.**	**ref.**	**ref.**	**ref.**	**ref.**	**ref.**
200–299	−0.20 (−1.55, 1.15)	−0.27 (−1.59, 1.06)	−0.67 (−2.96, 1.61)	−0.60 (−2.87, 1.67)	5.82 (−2.80, 14.4)	6.83 (−1.32, 15.0)
300–399	−0.30 (−1.62, 1.02)	−0.70 (−2.00, 0.60)	−0.96 (−3.20, 1.28)	−1.36 (−3.61, 0.88)	1.49 (−6.94, 9.92)	2.67 (−5.39, 10.7)
400–499	−0.96 (−2.30, 0.37)	−0.92 (−2.24, 0.41)	−1.29 (−3.55, 0.98)	−1.09 (−3.36, 1.19)	5.41 (−3.13, 14.0)	7.51 (−0.66, 15.7)
≥500	−0.76 (−1.96, 0.43)	−0.94 (−2.14, 0.27)	−1.40 (−3.42, 0.63)	−1.52 (−3.59, 0.55)	0.83 (−6.78, 8.45)	3.84 (−3.60, 11.3)
**Viral load suppression (<1000 copies/mL)**						
Yes	**ref.**	**ref.**	**ref.**	**ref.**	**ref.**	**ref.**
No	0.18 (−3.12, 3.47)	−0.19 (−3.42, 3.03)	−0.61 (−6.19, 4.98)	−1.16 (−3.71, 4.39)	−10.5 (−31.6, 10.5)	−1.21 (−21.1, 18.6)
**Haemoglobin (ug/dL)**						
≥10.0	**ref.**	**ref.**	**ref.**	**ref.**	**ref.**	**ref.**
<10.0	−1.71 (−4.10, 0.67)	−1.18 (−3.52, 1.16)	−2.70 (−6.74, 1.34)	−2.19 (−6.22, 1.84)	−6.23 (−21.5, 9.00)	−0.56 (−15.0, 13.9)
**Sex**						
Male	**ref.**	**ref.**	**ref.**	**ref.**	**ref.**	**ref.**
Female	0.87 (0.28, 1.46)	0.16 (−0.46, 0.78)	1.22 (0.21, 2.21)	0.42 (−0.65, 1.49)	−5.03 (−9.03, −1.15)	−3.96 (−7.80, −0.12)
**Nucleoside reverse transcriptase inhibitor**						
Lamivudine	**ref.**	**ref.**	**ref.**	**ref.**	**ref.**	**ref.**
Emtricitabine	−0.74 (−1.41, −0.08)	−0.30 (−1.11, 0.51)	−0.83 (−1.96, 0.30)	−0.01 (−1.40, 1.39)	0.67 (−3.59, 4.92)	0.12 (−4.87, 5.11)
**Time on ART (months)**						
0–11.9	2.65 (1.66, 3.65)	2.32 (1.23, 3.41)	4.20 (2.51, 5.89)	3.98 (2.11, 5.86)	−0.36 (−6.79, 6.08)	3.25 (−3.48, 9.98)
12–23.9	0.51 (−0.31, 1.32)	0.07 (−0.86, 1.00)	0.53 (−0.86, 1.91)	0.24 (−1.36, 1.84)	−2.12 (−7.39, 3.14)	1.05 (−4.69, 6.78)
24–35.9	−0.04 (−1.00, 0.92)	−0.06 (−1.08, 0.95)	0.02 (−1.60, 1.65)	0.14 (−1.61, 1.89)	−1.20 (−7.38, 4.97)	2.65 (−3.63, 8.93)
36–47.9	0.19 (−0.88, 1.26)	0.06 (−1.07, 1.20)	0.38 (−1.44, 2.21)	0.42 (−1.54, 2.37)	−2.57 (−9.51, 4.36)	−2.46 (−9.49, 4.57)
48–59.9	−0.08 (−1.18, 1.03)	−0.18 (−1.34, 0.99)	−0.33 (−2.21, 1.54)	−0.26 (−2.27, 1.75)	2.22 (−4.91, 9.36)	2.05 (−5.17, 9.26)
≥60	**ref.**	**ref.**	**ref.**	**ref.**	**ref.**	**ref.**
**Body Mass Index (kg/m**^**2**^**)**						
<18.5	1.23 (−0.54, 3.00)	1.08 (−0.68, 2.83)	2.74 (−0.30, 5.77)	2.48 (−0.54, 5.51)	2.20 (−9.33, 13.7)	3.34 (−7.52, 14.2)
18.5–24.9	2.84 (1.05, 4.63)	2.86 (1.08–4.65)	4.94 (1.87, 8.01)	4.94 (1.87, 8.02)	11.7 (0.03, 23.3)	9.78 (−1.24, 20.8)
25–29.9	4.22 (2.44, 5.99)	4.27 (2.48, 6.06)	6.08 (3.04, 9.12)	6.13 (3.04, 9.21)	9.61 (−1.94, 21.2)	8.20 (−2.87, 19.3)
≥30.0	**ref.**	**ref.**	**ref.**	**ref.**	**ref.**	**ref.**
**Hypertension**						
No	**ref.**	**ref.**	**ref.**	**ref.**	**ref.**	**ref.**
Yes	−0.87 (−1.67, −0.06)	−1.03 (−1.83, 0.24)	−1.13 (−2.49, 0.22)	−1.33 (−2.70, 0.04)	30.7 (25.8, 35.6)	26.8 (21.9, 31.7)

∗ Models for the mean change and weight and percent change in weight are also adjusted for continuous days between date of baseline weight and time of switch and days between follow-up weight and 12-months. For the outcome of hypertension, the complete list of variables in the table were controlled for in the model.

### Association of switching from efavirenz to dolutegravir on hypertension at 12 months

We saw an increased risk of hypertension at 12 months when comparing dolutegravir exposed to dolutegravir unexposed patients (risk difference (RD): 14.2% 95% CI: 10.6, 17.7%) (Table 3). We also found that patients with hypertension (vs. not) at time of switch (RD: 26.8%; 95% CI: 21.9, 31.7%) had an increased risk of hypertension over 12 months. Additionally, females (vs. males) in our cohort had a 3.96 percentage point decrease in the risk of hypertension (RD: −3.96%; 95% CI: −7.80, −0.12)

### Effect measure modification

This study was not powered to identify effect modifications, as such, results of this analysis should be interpreted as hypothesis-generating (Table 4). We assessed effect modification (interaction) between dolutegravir exposure and the outcome of mean change in weight by adding interaction terms between dolutegravir and all variables, separately, in our model. We saw the largest mean change in weight among those on dolutegravir and the following covariates; older age, BMI in the pre-obese and obese categories, hypertension, virally suppressed, higher haemoglobin levels, on a regimen containing lamivudine and being on ART ≥24 months. Although we saw similar weight gain in women compared to men on dolutegravir (mean change in males 1.45 kg; 95% CI: 0.61, 2.29 kg; mean change in females 1.24 kg; 95% CI: 0.41, 2.06 kg) (comparison between rows in Table 4), within exposure groups we found that women had a higher mean change in weight compared to their male counterparts (efavirenz group 0.98 kg; 95% CI: 0.25, 1.71 kg; dolutegravir group 0.77 kg; 95% CI: −0.16, 1.79) (comparison within columns in Table 4).

**Table 4:** Effect measure modification for mean change in weight from baseline to 12-months.

Stratification variables	Mean change in weight (kg)		
	Efavirenz mean (95% CI)	Dolutegravir mean (95% CI)	Crude mean change between columns (95% CI)
**Original analysis (crude)**	1.45 (SD 5.24)	2.79 (SD 6.66)	1.34 (0.75, 1.93)
**Sex**			
Male	0.97 (0.39, 1.56)^a^	2.42 (1.84, 3.00)	1.45 (0.61, 2.29)
Female	1.95 (1.36, 2.55)	3.19 (2.59, 3.79)	1.24 (0.41, 2.06)
**Age (years)**			
16–24.9	1.45 (0.35, 2.55)	1.72 (0.56, 2.87)	0.27 (−1.73, 2.27)
25–29.9	2.50 (0.88, 4.13)	2.68 (1.10, 4.26)	0.18 (−1.96, 2.31)
30–39.9	1.84 (1.14, 2.54)	2.61 (1.89, 3.33)	0.77 (−0.14, 1.68)
40–49.9	1.35 (0.59, 2.12)	3.30 (2.56, 4.03)	1.94 (0.85, 3.03)
≥50	0.24 (−0.86, 1.33)^a^	3.11 (2.02, 4.21)	2.88 (1.42, 4.34)
**CD4 count (cells/mm**^**3**^**)** (%)			
<200	2.32 (0.57, 3.97)^a^	3.00 (1.52, 4.48)	0.28 (−1.76, 3.12)
200–299	1.69 (0.60, 2.79)	3.26 (2.19, 4.33)	1.57 (0.10, 3.03)
300–399	2.04 (1.04, 3.05)	2.74 (1.73, 3.75)	0.70 (−0.66, 2.06)
400–499	1.45 (0.41, 2.49)	2.03 (0.97, 3.10)	0.59 (−1.50, 2.67)
≥500	1.03 (0.42, 1.65)	2.88 (2.25, 3.50)	1.84 (1.10, 2.86)
**Viral load suppression (<1000 copies/mL)**			
No	3.51 (−0.93, 7.96)^a^	0.88 (−3.92, 5.68)	−2.63 (−8.73, 3.47)
Yes	1.43 (1.01, 1.85)	2.81 (2.39, 3.23)	1.37 (0.78, 1.97)
**Time on ART (months)**			
0–11.9	4.15 (2.92, 5.38)	4.60 (3.36, 5.83)	0.44 (−1.50, 2.40)
12–23.9	2.07 (1.16, 2.97)	2.40 (1.44, 3.36)	0.33 (−0.98, 1.64)
24.0–35.9	0.23 (−0.94, 1.39)	3.15 (1.98, 4.32)	2.92 (1.57, 4.27)
36.0–47.9	1.15 (−0.23, 2.53)	2.60 (1.28, 3.93)	1.45 (−0.24, 3.15)
48–59.9	0.20 (−1.22, 1.62)	3.00 (1.61, 4.38)	2.80 (1.06, 4.54)
≥60	1.06 (0.39, 1.74)^a^	2.35 (1.69, 3.01)	1.28 (0.27, 2.30)
**Body Mass Index** (kg/m^2^)			
<18.5	−0.55 (−3.06, 1.95)^a^	−0.50 (−2.80, 1.79)	0.05 (−1.55, 1.64)
18.5–24.9	0.11 (−0.57, 0.80)	1.28 (0.60, 1.96)	1.17 (0.31, 2.02)
25–29.9	1.51 (0.73, 2.30)	3.08 (2.31, 3.84)	1.56 (0.53, 2.59)
≥30.0	2.91 (2.22, 3.60)	4.53 (3.82, 5.24)	1.62 (0.45, 2.79)
**Haemoglobin (ug/dL)** (%)			
<10.0	−0.12 (−3.84, 3.60)	0.81 (−2.23, 3.84)	0.93 (−2.10, 3.95)
≥10.0	1.47 (1.05, 1.89)^a^	2.83 (2.41, 3.25)	1.36 (0.76, 1.96)
**Nucleoside reverse transcriptase inhibitor**			
lamivudine	1.57 (1.08, 2.06)^a^	3.07 (2.57, 3.55)	1.50 (0.85, 2.14)
emtricitabine	1.14 (0.35, 1.93)	2.04 (1.24, 2.85)	0.90 (−0.42, 2.22)
**Hypertension** (%)			
No	1.68 (1.23, 2.14)^a^	2.85 (2.39, 3.30)	1.17 (0.53,1.80)
Yes	0.25 (−0.79, 1.29)	2.51 (1.49, 3.53)	2.26 (0.68, 3.85)

a Reference group.

## Discussion

In this observational study of a predominately black African ancestry population, we assessed the association of dolutegravir on weight change in an adult population of PLWH. Over a 12-month follow-up period, we found a greater mean and percentage increase in weight among patients who switched from efavirenz to dolutegravir (exposed) compared to patients who remained on efavirenz (unexposed). These results are in line with previously published literature, which suggest that non-nucleoside reverse transcriptase inhibitors (NNRTIs), such as efavirenz, are associated with lower average weight gain than are INSTIs, such as dolutegravir.^15^^,^^16^

A number of studies have now evaluated dolutegravir specifically, given its potential differential effect on weight. Previous studies observed weight increases of 3–4 kg among those on dolutegravir,^17^^,^^18^ and a post hoc analysis of the NEAT-002 study showed a significant, albeit small, weight gain after switching to dolutegravir from a ritonavir-boosted protease inhibitor.^19^ Further compelling data comes from the NAMSAL and ADVANCE trials. In both studies, participants randomized to dolutegravir gained more weight on average than those on efavirenz.^7^^,^^8^^,^^20^ Moreover, in the ADVANCE study, dolutegravir + emtricitabine/tenofovir alafenamide was independently associated with ≥10% increase in body weight and treatment-emergent obesity. More recently, out of the African Cohort Study (AFRICOS), which was also conducted in routine care settings, researchers found the average change in weight prior to switching to a dolutegravir-based regimen was 0.35 kg/year, which increased to an average change in weight of 1.46 kg/year in the year following transition to dolutegravir, corresponding to a 4-fold increase.^21^ Our results provide support for these findings.

Prior literature has also demonstrated potential differential effects of INSTIs on weight gain among men and women.^21^^,^^22^ In the NAMSAL study, women were more likely to experience a ≥10% change in weight over 48-week follow-up period compared to men.^23^ In a multinational cohort of adults with well-controlled HIV, women on dolutegravir had a higher mean BMI and waist circumference than did women on an alternative ART regimen or men on dolutegravir.^21^^,^^22^ Our results do not fully support this, as women in our cohort who switched to dolutegravir experienced similar weight gain as did men who switched to dolutegravir. This could be a result of a slightly higher uptake of dolutegravir in men compared to women in the full sample prior to matching. However, sex was balanced between efavirenz and dolutegravir groups in our final matched analytical sample, so we anticipate that this did not impact our study results. Our results did show that, within exposure groups, women had a higher mean change in weight compared to their male counterparts, potentially supporting the hypothesis that higher circulating leptin levels and subcutaneous adipose tissue metabolic rates among women may, in part, contribute to greater weight gain compared to men.^24^^,^^25^ Despite initial concerns regarding the teratogenicity of dolutegravir, including initial recommendations in the South African guidelines requiring explicit consent from women acknowledging this risk, the uptake of dolutegravir between men and women was equal in our study.

Additional data suggests that efavirenz itself may be the modifying factor, mitigating weight gain among slow metabolizers of the drug. Data from ADVANCE strongly suggests this is the case, reporting that weight gain among moderate and fast metabolizers of efavirenz was the same as that among those on the dolutegravir arm, containing the same nucleoside backbone.^26^^,^^27^ This is supported by other studies on the impact of efavirenz concentrations on weight gain.^28^

Results of this analysis, coupled with a large body of existing evidence, establish a clear association between INSTIs and weight gain, a key risk factor for hypertension, though the data linking these agents with hypertension is mixed. Clinical trials have reported no increased risk of hypertension,^29^^,^^30^ while analyses from observational cohorts, including our own, have reported a higher risk of hypertension or elevated blood pressure in PLWH using INSTIs.^31^, ^32^, ^33^, ^34^ There are a number of hypothesized pathways for this increased risk, primarily mediated through changes in BMI,^33^ however INSTI use has also been linked to dyslipidaemia, adipogenesis, oxidative stress and insulin resistance, all of which have been associated with hypertension.^32^ Though previous studies have also linked our comparative agent, efavirenz, to dyslipidaemia,^35^ it was not shown to be associated with an increased risk of hypertension.^36^ Further research is needed to delineate the metabolic pathway between INSTI use and hypertension.

A few limitations must be considered when interpreting the results of this study. First, because our study reports on a single government HIV clinic that almost exclusively provides services to patients of black African ancestry, our results may not be generalizable to other populations. Second, although we offer compelling data from a large, well-balanced cohort, the observational nature of this study inhibits us from making conclusions about a causal relationship. Third, we do not have data on why some patients were switched to dolutegravir while others were maintained on efavirenz. Fourth, while access to a well-established data system like TherapyEdge-HIV™ allowed us to use propensity score matching to account for multiple measured confounding variables, it does not account for potentially unmeasured confounders. Fifth, we found that pre-obese and obese patients had a higher likelihood of switching from efavirenz to dolutegravir. If these individuals had a higher likelihood of being switch to dolutegravir, our results could be biased. However, BMI groups were balanced between efavirenz and dolutegravir groups in our final matched analytical sample, so we anticipate that this did not impact our study results. Sixth, we were also unable to account for slow metabolizers of efavirenz or dolutegravir, other drugs associated with weight gain (e.g. psychotropic drugs), or behavioural factors such as alcohol or recreational drug use and physical activity. Seventh, our definition of hypertension is partly reliant upon a single blood pressure measurement and therefore may overestimate the true association in our population. Finally, it is important to note that follow up time was relatively short and may not be considered adequate in evaluating cardiovascular endpoints.

Our data confirm findings from previous clinical trials and demonstrate that in a real-world population of PLWH on ART, patients gained 1.78 kg in weight 12-months after switching from efavirenz to dolutegravir than those remaining on efavirenz. We also saw an increase in the risk of hypertension over 12-months. Longer follow-up is needed, however, to determine if INSTI-associated weight gain is associated with changes in NCD risk over the long-term, or whether weight gain is sustained, as seen in clinical trials.

## Contributors

All authors were involved in the design of the study. AB and CN accessed and verified the data. AB did the statistical analysis with support from CN. AB and CN wrote the first draft of the article. AB, CN, WDFV, EK, SR, MM, AS and MF have full access to all the data in the study. All authors critically interpreted the results and developed the report. All authors reviewed and approved the final version.

## Data sharing statement

The data in our study are not publicly available due to the terms of our contract with the Right to Care.

## Declaration of interests

Emma Kileel (EM) has received contractor fees from Massachusetts General Hospital for work unrelated to the present manuscript. Andrew Stokes (AS) has received research support from Johnson & Johnson, Inc. For work unrelated to the present manuscript. Willem DF Venter's (WDFV) unit receives funding from the 10.13039/100000865Bill and Melinda Gates Foundation, SA 10.13039/501100000265Medical Research Council, National Institutes for Health, Unitaid, Foundation for Innovative New Diagnostics (FIND) and the Children's Investment Fund Foundation (CIFF). WDFV has previously received funding from USAID, and receives drug donations from ViiV Healthcare, Merck, J&J and Gilead Sciences for investigator-led clinical studies. WDFV's unit does investigator-led studies with Merck, J&J and ViiV providing financial support and is doing commercial drug studies for Merck. WDFV's unit performs evaluations of diagnostic devices for multiple biotech companies. Individually, WDFV receives honoraria for educational talks and advisory board membership for Gilead, ViiV, Mylan/Viatris, Merck, Adcock-Ingram, Aspen, Abbott, Roche, J&J, Sanofi and Virology Education. All other authors declare no competing interests.
