# Supplementary material for: Change in body weight and risk of hypertension after switching from efavirenz to dolutegravir in adults living with HIV: evidence from routine care in Johannesburg, South Africa
Source: eClinicalMedicine. 2023 Feb 6;57:101836. doi: 10.1016/j.eclinm.2023.101836 (PMC9932660; doi:10.1016/j.eclinm.2023.101836)
Supplement: Supplemental Fig. S1 [file mmc1.docx]

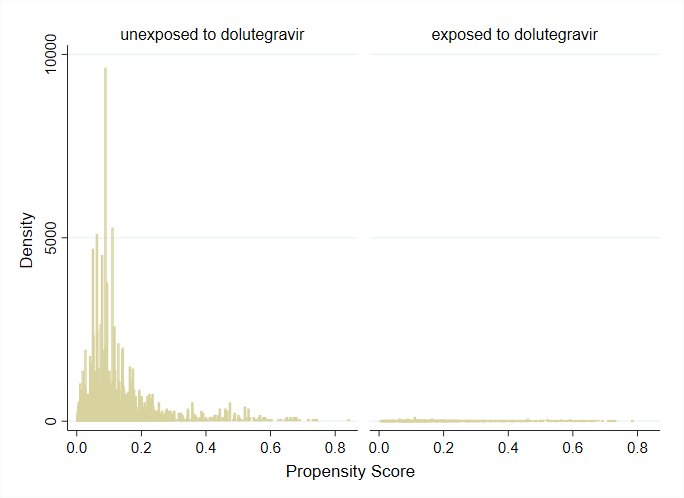
**Supplemental Figure 1. Histogram of propensity scores of unexposed and exposed patients used to perform the matching among stable patients on HIV treatment at the Themba Lethu Clinic in Johannesburg South Africa (n=6,948).**
